# Case report: Is bilateral renal dioctophymosis and severe uremia in a dog synonymous of euthanasia? Not today

**DOI:** 10.3389/fvets.2024.1396467

**Published:** 2024-05-22

**Authors:** Pâmela Caye, Jean Carlos Gasparotto, Anna Vitória Hörbe, Letícia Rodrigues Leitao Andrade, Rainer da Silva Reinstein, Luiza Tonietto Mangini, Daniel Curvello de Mendonça Müller, Maurício Veloso Brun

**Affiliations:** ^1^Postgraduate Program in Veterinary Medicine, Center for Rural Sciences, Federal University of Santa Maria, Santa Maria, Brazil; ^2^Residency Program in Veterinary Medicine, University Veterinary Hospital, Federal University of Santa Maria, Santa Maria, Brazil; ^3^Degree in Veterinary Medicine, Center for Rural Sciences, Federal University of Santa Maria, Santa Maria, Brazil; ^4^Department of Small Animal Clinic, Rural Science Center, Federal University of Santa Maria, Santa Maria, Brazil; ^5^Brazilian National Council for Scientific and Technological Development (CNPq) Research Productivity Scholarship-Level 1C (3304353/2021-3), Brazil

**Keywords:** dioctophymosis, *Dioctophyme renale*, giant kidney worm, nephroscopy, hyperbaric oxygen therapy

## Abstract

A dog with bilateral renal dioctophymosis presented with stage 5 acute kidney injury, weight loss, vomiting, apathy, and hematuria. Laboratory tests showed creatinine of 17.2 mg/dL and *Dioctophyme renale* eggs in the urine. It underwent a 30-min session of hyperbaric oxygen preconditioning at a pressure of 2 ATA. Subsequently, bilateral nephroscopy was performed, without warm ischemia, using Amplatz-type renal dilators. Five parasites were removed, three females from the right kidney, one female from the left kidney, and one male from the abdominal cavity. After surgery, the patient continued doing daily hyperbaric oxygen therapy (HBOT) sessions and clinical therapy. Postoperative care consisted of analgesics, antimicrobials, antioxidants, gastric protector and fluid therapy. Ultrasound monitoring showed a reduction in the area of renal dilation and the hematological and biochemical tests showed rapid recovery from acute kidney injury. There was no bacterial growth in the urine sample collected directly from the kidneys. The patient had an excellent clinical progression and was discharged from hospital 7 days postoperatively, with creatinine values of 2.9 mg/dL. This is the first report of the use of nephroscopy in the treatment of dioctophymosis and indicates excellent chances of cure even in severe cases of bilateral parasitosis. HBOT was shown to be an ally in the clinical therapy of patients with *D. renale* by helping with stabilization and postoperative recovery.

## Introduction

Dioctophymosis in dogs occurs due to parasitism by *Dioctophyme renale*, a cosmopolitan nematode with worldwide distribution and common in southern Brazil (1–4). The parasite usually affects the hosts' right kidney, leading to chronic and irreversible destruction of the renal parenchyma (3, 5–8). Although rare, there are reports of bilateral renal dioctophymosis in dogs (9, 10) and humans (11), with most cases associated with patient death. Treatment is surgical and usually involves nephrectomy of the parasitized kidney (8). In general, nephron-sparing surgery is recommended, especially when there is major impairment of renal function and some renal viability. There are reports of laparoscopic nephrotomy to remove parasites in dogs with unilateral dioctophymosis (12). To the authors' knowledge, there are no reports of nephroscopy for the treatment of parasitosis. This report describes the first bilateral nephroscopy for the treatment of a dog with *D. renale* presenting with severe acute kidney injury (AKI).

## Case description

A greyhound female dog, estimated to be 5 years old, was treated at a veterinary hospital after being referred by another professional. Six days before, it had been diagnosed with bilateral renal dioctophymosis due to *D. renale*. The owner reported that, at the time of diagnosis, it had been losing weight and appetite, showing apathy and its urine was red. On what was established as Day 1, the veterinarian responsible for the referral ordered hematological tests, which showed no abnormalities, and renal biomarkers, which indicated severe azotemia (Table 1). Urinalysis showed leukocyturia, hematuria, proteinuria, and *D. renale eggs*. It is noteworthy that creatinine had been measured 15 days previously and was slightly elevated (1.78 mg/dL).

**Table 1:** Laboratory tests performed before and after nephroscopy and hyperbaric oxygen therapy in a dog with bilateral *Dioctophyme renale*.

**Test**	Day 1^*^	Day 3^**^	**Day 4**	**Day 5**	**Day 6**	**Day 7**	**Day 10**	**Day 22**	**Day 61**
**Hematology**									
Red blood cells (10^6/^mm^3^)	6.75	6.23	4.73	4.19	-	4.39	4.17	4.9	6.6
Hemoglobin (g/dL)	12.8	13.3	9.6	9.1	-	10	9.3	11.6	14.3
Hematocrit (%)	40	38.3	26.7	26.1	-	27.9	27.3	35	43
TPP (g/dL)	7.8	8.2	7.2	7	-	7.4	7.6	-	-
Platelets (/μL)	281,000	208,000	255,000	281,000	-	297,000	414,000	345,000	224,000
Total leukocytes (/μL)	17,600	13,200	13,800	16,200	-	10,900	15,500	10,500	11,000
Segmented (/μL)	10,736	7,788	10,212	3,968	-	4,033	10,385	4,200	5,720
Eosinophils (/μL)	2,640	2,112	966	1,620	-	2,289	1,085	2,625	0
Lymphocytes (/μL)	3,168	2,112	2,208	558	-	3,270	3,255	3,360	4,840
Monocytes (/μL)	1,056	1,188	414	2,754	-	1,308	775	315	440
**Serum biochemistry**									
Creatinine (mg/dL)	11.02	17.9	15.3	11.5	7.8	5	2.9	2.11	-
Urea (mg/dL)	222.9	284	288	255	211	143	101	33.7	-
Phosphorus (mmol/L)	9.52	-	-	-	6.5	-	3.4	3.52	4.39
Potassium (mmol/L)	5.1	-	-	-	3.96	-	-	-	-
Sodium (mmol/L)	146	-	-	-	152	-	-	-	-
SDMA (μg/dL)	32	-	-	-	-	-	-	-	-
**Urinalysis**									
Urine density	1,025	-	-	-	-	-	1,016	1,015	-
Leukocytes/field	10–20	-	-	-	-	-	0–2	0–1	-
Red blood cells/field	>100	-	-	-	-	-	10–20	0–1	-
Bacteria/field	++	-	-	-	-	-	+	+	-
*D. renale* eggs	Yes	-	-	-	-	-	No	-	-
Urinary UP/C	1.23	-	-	-	-	-	1.35	0.41	-

TPP, total plasma protein; SDMA, symmetric dimethylarginine; UP/C, urinary protein/creatinine ratio.

^*^Tests performed by another professional, 48 h before the patients was seen and whose results were sent together with the patient's referral.

^**^Surgical evaluation and treatment.

At the time of its visit to the University Veterinary Hospital from Federal University of Santa Maria on Day 3, the patient had been suffering from anorexia for 4 days, increased fluid intake, nausea, vomiting, pasty stools, hematochezia, and hematuria. The following was described on clinical examination: weight of 22.6 kg, body score of 2/5, dehydration of 6%, abdominal rigidity, pain on palpation, capillary refill time of 3 s. Hematological and biochemical tests and ultrasound were repeated. The results are presented in Table 1. Worsening of biomarkers of renal function was observed, characterizing the clinical picture as uremic syndrome and classifying it as non-oliguric stage 5 AKI, according to IRIS (13).

**Figure 1:**
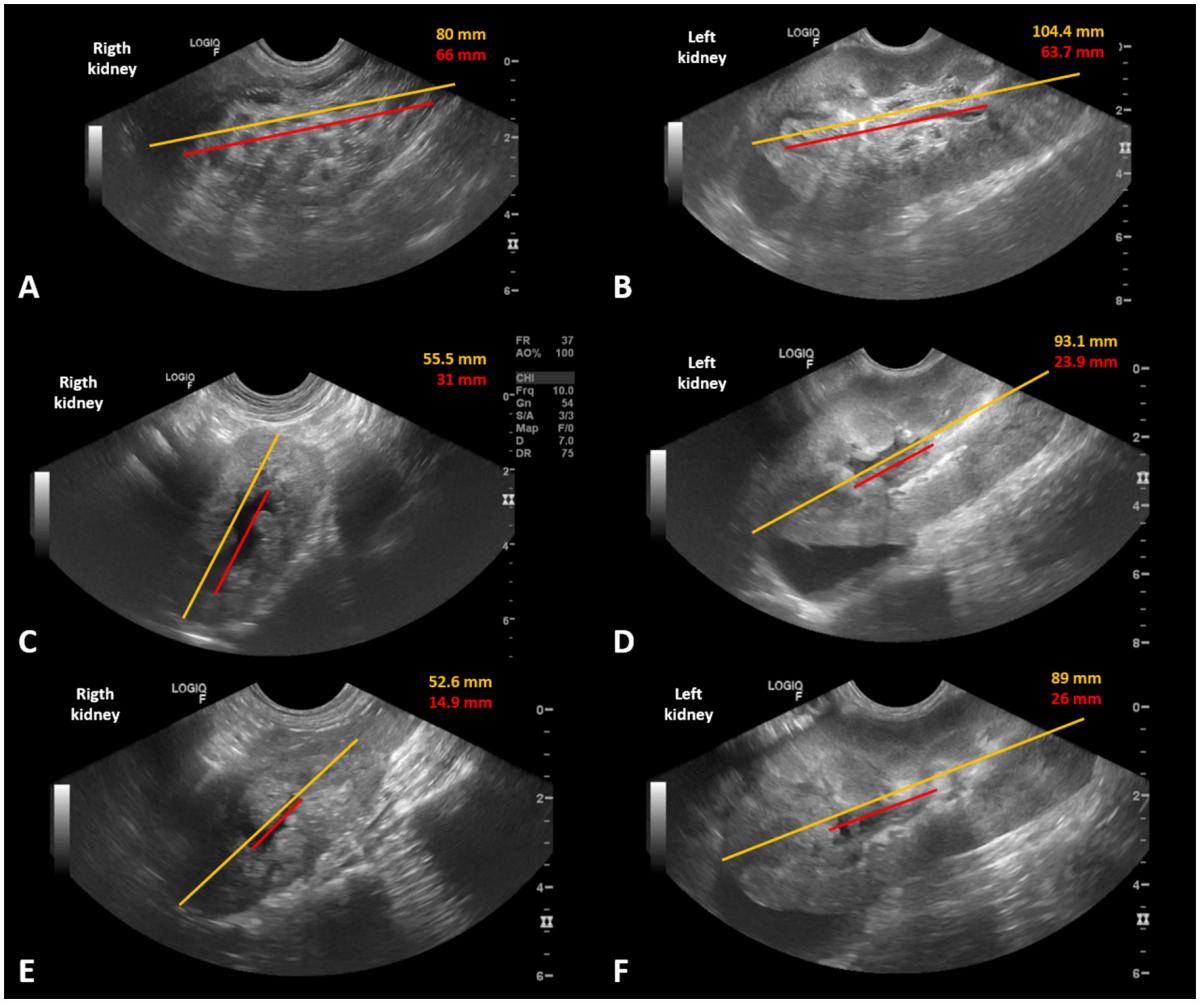
Ultrasound images of a dog with bilateral *Dioctophyme renale*, before and after bilateral nephroscopy to remove the parasites. **(A)** Preoperative appearance of the right kidney full of tubular structures with hyperechoic borders and a hypoechoic center. **(B)** Preoperative appearance of the left kidney, with the same structures, restricted to the internal medullary region and renal pelvis. **(C, D)** Appearance 48 h after the surgical procedure to remove the kidney parasites, with a significant reduction in the length and dilation of the bilateral pelvis. **(E, F)** Appearance 7 days after the surgical procedure, with a reduction in the hyperechogenicity of the parenchyma and inflammation, as well as a reduction in the dilation of both pelvises. Yellow line—approximate measurement of kidney length in a longitudinal section. Red line—approximate measurement of the area where the parasites were located and dilation of the pelvis.

Abdominal ultrasound confirmed the bilateral presence of parasites (Figures 1A, B). The right kidney measured 80 mm and had multiple cylindrical structures with hyperechoic edges and hypoechoic center. Minimal renal parenchyma was visualized. The area corresponding to the pelvis and the site where the parasites were found measured 66 mm. The left kidney was hypertrophic (104.4 mm) and had the same structures, although restricted to the internal medulla and renal pelvis (63.7 mm). The parenchyma appeared intact, with reduced corticomedullary definition and a diffuse increase in echogenicity. There was retroperitoneal reactivity adjacent to the renal contours.

**Figure 2:**
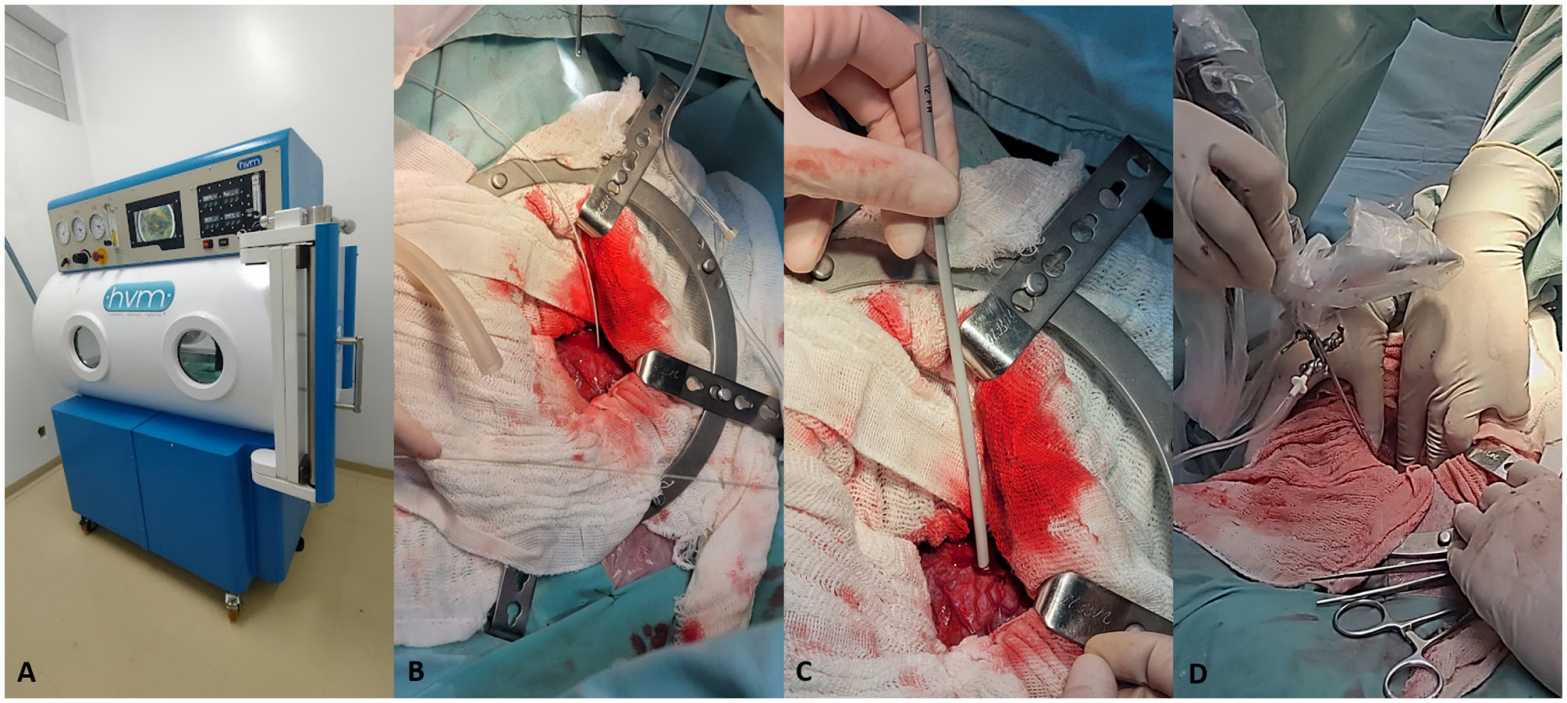
Hyperbaric oxygen therapy and bilateral nephroscopy performed in a dog with bilateral *Dioctophyme renale*. **(A)** Monoplace hyperbaric chamber (HVM™) during therapy at 2 ATA pressure. **(B)** Left kidney with guidewire positioned inside the kidney, after puncture with a 14G catheter. **(C)** Dilation of the orifice with an Amplatz-type 12 Fr renal dilator. **(D)** Nephroscopy with a 9.6 Fr rigid cystoscope and irrigation of the renal pelvis with sterile solution.

The patient immediately began treatment with fluid therapy with Ringer's lactate (4 ml/kg/h) to correct dehydration, analgesia with dipyrone (25 mg/kg, IV) and methadone (0.2 mg/kg, SC), and antibiotic therapy with cephalothin (30 mg/kg, IV). After administration of the medications, the patient underwent the first session of hyperbaric oxygen therapy (HBOT; Figure 2A). The therapy was carried out in a veterinary monoplace chamber (HVM™), pressurized with 100% oxygen at a pressure of 2 ATA (atmosphere absolute). The dog remained inside the chamber for 60 min, including 15 min of pressurization, 30 min of therapy, and 15 min of depressurization. It made a slight rapid head movement, suggesting ear discomfort, and urinated inside the chamber. It did not show any other type of discomfort during the session and remained lying down and calm.

It then underwent a bilateral nephroscopy surgical procedure. Anesthesia induction was performed using ketamine (1 mg/kg), fentanyl (3 μg/kg), and propofol (5 mg/kg), IV. After orotracheal intubation and stabilization of the anesthetic plane, epidural anesthesia was performed with 0.5% bupivacaine (0.33 ml/kg) associated with morphine (0.1 mg/kg) and fentanyl (2 μg/kg). Ketamine (0.6 mg/kg/h) and dexmedetomidine (0.5 mg/kg/h) were administered in continuous infusion to reduce the minimum alveolar concentration (MAC) of the halogenated anesthetic (isoflurane) and provide analgesia. The patient was positioned in the supine position and the surgical site was prepared aseptically. Pre-umbilical celiotomy was performed and a wound retractor ring was placed. Diffuse reactivity was detected in the omentum, as well as the presence of a free 20-cm male parasite, close to the left kidney.

**Figure 3:**
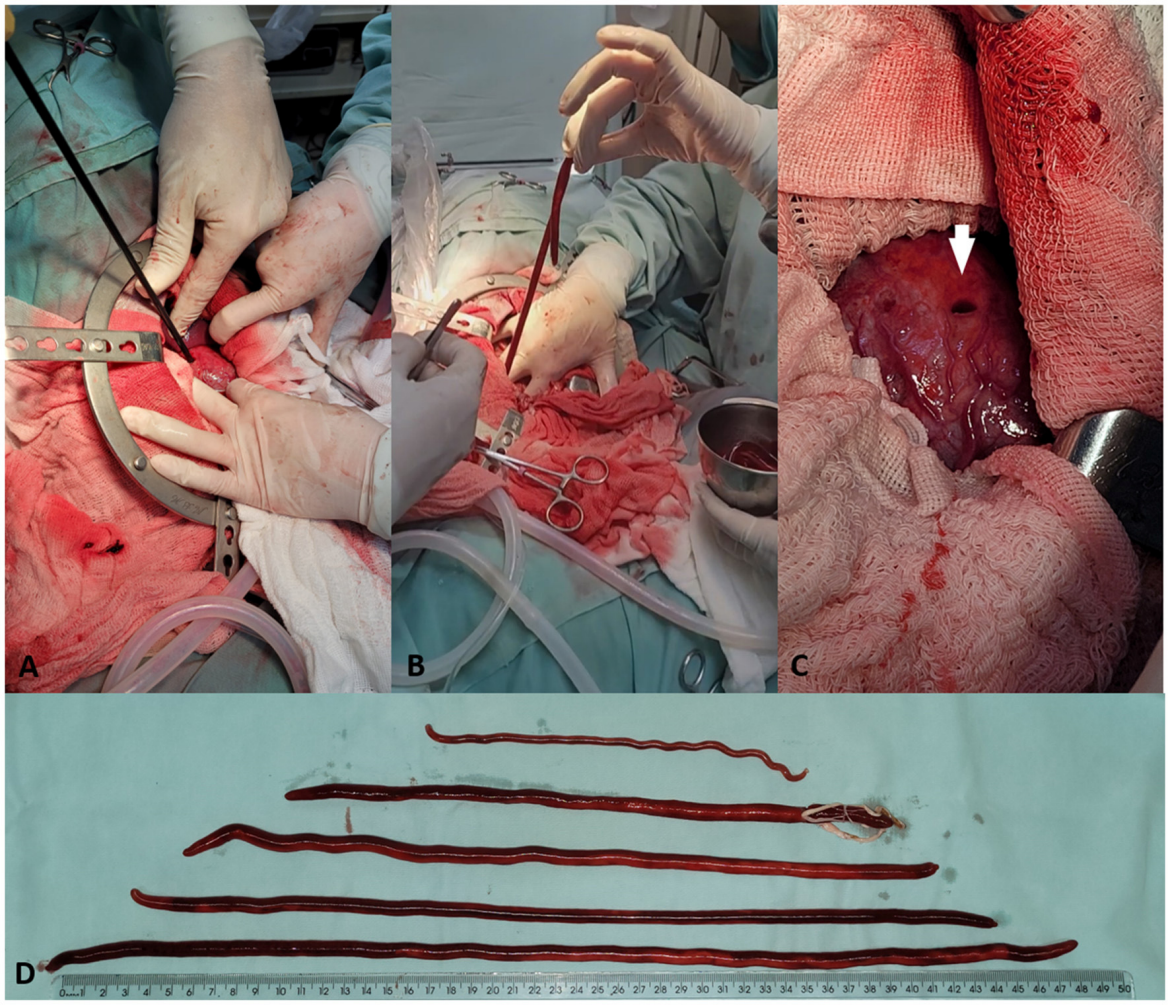
Bilateral nephroscopy to remove *Dioctophyme renale* in a dog. **(A)** Insertion of 5-mm laparoscopic Kelly forceps to capture the parasite. **(B)** Extraction of a female parasite removed from the inside of the right kidney. **(C)** Final appearance of the 16 Fr orifice (white arrow) through which the parasites were removed from the kidney interior. **(D)** One male (above) and five females of *D. renale* removed from a parasitized dog.

The left kidney showed hypertrophy and increased vascular pattern. It was punctured in the medial region with a 14 Fr catheter, until urine was obtained, and a hydrophilic guidewire was inserted (Figure 2B). Then, Amplatz-type renal dilators 10, 12, 14, and 16 Fr were used progressively (Figure 2C). Renal artery hemostasis was not performed and there was no relevant hemorrhage. Nephroscopy was performed with a 30-degree and 1.9–2.2 mm diameter telescope (Karl Storz™) positioned in a 9.6 Fr cystoscopy sheath. After the infusion of saline solution, a parasite was visualized that completely obstructed the ureter (Figure 2D). Cystoscopy grasping forceps and DeBakey atraumatic forceps were not sufficient for traction, and 5-mm laparoscopic Kelly forceps were used to remove a 33-cm female parasite (Figures 3A, B). The renal pelvis was inspected and cleaned. The 16 Fr hole (Figure 3C) was closed with a 3-0 polydioxanone suture in a horizontal mattress pattern.

The right kidney had an altered conformation, smaller than the left kidney. The same access technique was used but there was difficulty in capturing the parasites because they were in the pelvic recesses. The same kind of laparoscopy forceps were used and one parasite was removed. On palpation it was suspected that there were more parasites, but the forceps did not reach any more. Reinspection and washing with a cystoscope advanced toward the cranial renal pole allowed more specimens to be found and two more females were removed. In total, the right kidney had three female parasites measuring 38, 42, and 50 cm (Figure 3D). Reinspection confirmed a parasite-free renal interior, which was irrigated with saline solution and sutured with the same technique used in the contralateral kidney.

On first inspection, the left kidney did not present the same interior image as the right kidney. Therefore, to ensure the removal of all parasites, the first renal suture was undone. The kidney was reevaluated to confirm the absence of parasites and sutured again. The abdominal cavity was irrigated with sterile solution and the two renal access points were omentalized. Routine three-layer closure of the incision was performed. Next, an esophageal feeding tube was placed.

Postoperative care consisted of analgesics, antimicrobials, antioxidants, gastric protector, and other medications to aid the patient's recovery. The medications used were as follows: methadone 0.3 mg/kg SC q6h for 24 h, followed by 0.2 mg/kg SC q6h for the following 24 h; tramadol 4 mg/kg IV q8h, started after methadone suspension and used for 3 days; dipyrone 25 mg/kg IV q8h for 7 days; cephalothin 30 mg/kg IV q8h for 5 days; maropitant citrate 1 mg/kg IV q24h for 4 days; omeprazole 1 mg/kg IV q12h for 4 days, later replaced by oral omeprazole, same dose, administered every 24 h for another 3 days; aluminum hydroxide 20 mg/kg, PO q12h for 7 days; probiotic paste 4 g/VO q24h; ferrous sulfate 120 mg/animal PO q24h for 7 days and omega-3 1,000 mg/animal PO q24h in continuous use.

Eighteen hours after the end of the surgical procedure, the HBOT sessions were resumed, using the protocol described previously, at 24-h intervals, totaling four postoperative sessions. Fluid therapy was performed with Ringer's lactate at a dose of 4 ml/kg/h during the first 24 h after surgery, during which time urine production was 2.8 ml/kg/h. Then, because the patient also received hydration via an esophageal tube, fluid therapy was reduced to a rate of 2 ml/kg/h and kept for 3 days. The animal was fed through an esophageal tube until it gradually started eating spontaneously, approximately on the fifth postoperative day.

Hematological and biochemical tests were performed daily and confirmed the rapid and satisfactory recovery from AKI. Serial abdominal ultrasounds confirmed the removal of all parasites and renal recovery. Figures 1A–F shows kidney size and the proportion of pelvic dilation in both kidneys. The measurements obtained were subjective because the kidney size exceeded the capacity of the ultrasound device to capture each organ in its entirety. A gradual bilateral reduction in the size and dilation of the renal pelvis was observed.

The severely parasitized right kidney measured 52.6 mm 7 days postoperatively, with loss of corticomedullary definition, irregular contours, and hyperechogenic heterogeneous parenchyma. There was slight dilation of the renal pelvis due to anechoic content (14.9 mm). The left kidney measured 82.6 mm, showed decreased corticomedullary definition due to diffuse increase in renal echogenicity and some hyperechogenic cortical striations, with preserved vascular flow in color Doppler mode. There was also slight dilation of the renal pelvis due to anechoic content (26 mm).

The patient presented normal physiological parameters for the species during the recovery period. With recovery, the medications and fluid therapy were reduced or discontinued. There was no bacterial growth in the urine sample collected directly from the kidneys. Clinical improvement was accompanied by normalization of behavior, including spontaneous water and food intake and active demeanor. The patient was discharged from hospital 7 days postoperatively, without an esophageal tube, with a prescription for kidney diet food, omega-3, and aluminum hydroxide. At this time, its condition was classified as stage 3 chronic kidney disease with proteinuria and prehypertension, according to IRIS (14).

The owner lives in a city far from the veterinary hospital (~290 km) where the animal was treated. Patient monitoring is carried out by a local nephrologist and by telephone. Reassessment 19 days postoperatively indicated mild anemia and increased creatinine. The patient continued to receive kidney food and omega-3 and was in excellent clinical condition 183 days after the surgical intervention.

## Discussion

Here we describe the therapeutic success of the first nephroscopy performed for the treatment of bilateral dioctophymosis in a dog. Minimally invasive surgical procedures have several benefits in dogs. However, they are also associated with ischemia/reperfusion (I/R) injuries caused by pneumoperitoneum and longer surgical times (15). Conventional incisional nephrotomy can temporarily reduce 25–50% of renal function (16). The traditional abdominal surgical approach was chosen by the surgeons considering the severity of the case, the presence of bilateral disease, and the need for reduced surgical time. Nevertheless, nephron-sparing techniques were used, with minimal kidney damage. Petrovsky et al. (17) described the use of nephroscopy for nephrolithotomy in a very similar way, which also made it possible to inspect the renal pelvis and confirm its complete emptying (17).

The use of dilators to access the renal pelvis, instead of the nephrotomy incision, significantly reduced intraoperative bleeding. Therefore, warm ischemia (WI) was not used in both kidneys. Studies have described the performance of partial nephrectomy without WI, which reduces the deterioration of the glomerular filtration rate (18). Raheem et al. indicated that renal WI longer than 30 min was not associated with long-term chronic kidney disease in human patients with two kidneys (19). However, WI is associated with renal I/R injury (20, 21) and the patient in the present case had severe bilateral renal involvement and grade 5 AKI. This explains the choice of faster and less harmful methods.

On the other hand, the limited access resulted in difficulty in inspecting the renal pelvis. Also, there are no descriptions of images from similar nephroscopy procedures to show the appearance after removing the parasites. Therefore, the first suture on the left kidney was removed to allow for a reevaluation of the organ, due to concerns about residual parasites. This could have been avoided with the use of intraoperative imaging tests, such as ultrasound, which was not available.

HBOT is a therapy that consists of delivering 100% oxygen in a pressurized environment. It is associated with improved healing response, synergy with antibiotics, reduction of vasogenic edema and treatment of gas embolism (22). It is well-tolerated by dogs and cats and has been applied for various purposes (23–25). At the renal level, both preconditioning and hyperbaric therapies after I/R injury showed induction of renal tissue protection. There was an improvement in the glomerular filtration rate and a reduction in urea and creatinine levels in rats (20, 26–28). HBOT has already been used in dogs with *D. renale* and the results were promising (29). Although there are no experimental studies with dogs, from the authors' experience, the patient's rapid improvement was a result of the hyperbaric preconditioning process and treatment continuation, associated with the appropriate postoperative clinical management.

Unilateral parasitosis is not usually associated with surgical emergencies, as shown in a study with 52 nephrectomy procedures in dogs with *D. renale* (8). However, this was a case of extremely severe dioctophymosis in a dog. The rapid and sudden progression of kidney injury is of note, with a 10-fold increase in creatinine within 20 days. The authors believe the severe progression of the disease was partly caused by unilateral ureteral obstruction caused by the left kidney parasite, which was resolved with removal of the worm. The patient's treatment was delayed, as there was a 6-day interval between diagnosis and surgery, as a result of the distance between the city of origin and the veterinary hospital that was capable of performing a procedure of this complexity. This interval also explains the serious progression of AKI. There is no doubt that without proper intervention, the patient would have quickly died.

There is still no drug therapy proven to be effective in curing dioctophymosis. Even if there were, the parasite causes pressure atrophy and necrosis of the renal parenchyma, which are accompanied by tissue inflammation (30). Therefore, killing the parasite without removing it would not prevent further kidney damage. The authors highlight here the importance of rapid surgical intervention to remove the parasites.

The most similar case in the literature is described by Borrelli (10), in which a dog underwent right nephrectomy and left nephrotomy using a conventional surgical technique. The current literature does not provide reports of similar cases for comparison of postoperative ultrasound images. However, the authors consider the imaging evolution of both kidneys very positive. Comparative serial measurements between total renal size and areas of pelvic dilation showed a progressive increase in the parenchyma/pelvis proportion. Kidney size reduction, hydronephrosis, and inflammatory appearance, associated with the increase in the proportion of renal parenchyma subjectively indicates renal function improvement.

The results presented here in allow us to conclude that bilateral nephroscopy, not associated with warm ischemia, leads to the cure of bilateral renal dioctophymosis when part of the renal parenchyma is viable. HBOT plays an important role in protecting renal function against parasitic, surgical, and inflammatory injury. This report offers a new perspective on the treatment of bilateral renal parasitosis in dogs, thus dispelling the idea that the disease inevitably leads to the death of severely parasitized patients.

The procedures performed in this case were part of the necessary emergency clinical treatment for the patient. Therefore, approval from the Institutional Animal Care and Use Committee was not required. Written informed consent was obtained from the owners for the participation of their animals in this study.

## Data availability statement

The original contributions presented in the study are included in the article/supplementary material, further inquiries can be directed to the corresponding author.

## Ethics statement

Written informed consent was obtained from the participant/patient(s) for the publication of this case report.

## Author contributions

PC: Conceptualization, Data curation, Writing – original draft, Writing – review & editing. JG: Data curation, Investigation, Methodology, Writing – original draft. AH: Data curation, Investigation, Methodology, Writing – original draft. LA: Data curation, Formal analysis, Writing – original draft. RR: Visualization, Writing – original draft. LM: Data curation, Investigation, Methodology, Writing – original draft. DM: Funding acquisition, Project administration, Resources, Writing – review & editing. MB: Investigation, Resources, Writing – review & editing.
